# Arterial site selection for measurement of mean arterial pressure in septic shock patients on high-dose norepinephrine

**DOI:** 10.3389/fmed.2022.1019752

**Published:** 2022-12-23

**Authors:** Bhanuprakash Bhaskar, Mohan Gurjar, Prabhaker Mishra, Afzal Azim, Banani Poddar, Arvind K. Baronia

**Affiliations:** ^1^Department of Critical Care Medicine, Sanjay Gandhi Postgraduate Institute of Medical Sciences (SGPGIMS), Lucknow, Uttar Pradesh, India; ^2^Department of Biostatistics and Health Informatics, Sanjay Gandhi Postgraduate Institute of Medical Sciences (SGPGIMS), Lucknow, Uttar Pradesh, India

**Keywords:** septic shock, invasive blood pressure, mean arterial pressure, hemodynamic monitoring, arteries, femoral-radial arterial pressure gradient, vasoconstrictor, norepinephrine

## Abstract

**Background:**

The guidelines of the Surviving Sepsis Campaign suggest using invasive blood pressure (IBP) measurement in septic shock patients, without specifying for a preferred arterial site for accuracy in relation to the severity of septic shock. The objective of this study was to determine the mean arterial pressure (MAP) gradient between the femoral and radial artery sites in septic shock patients.

**Method:**

This prospective study was carried out at a 20-bed ICU in a university hospital. Simultaneous MAP measurements at femoral and radial arterial sites were obtained in septic shock patients receiving norepinephrine (≥0.1 μg/kg/min), with a pre-planned subgroup analysis for those receiving a high dose of norepinephrine (≥0.3 μg/kg/min).

**Results:**

The median norepinephrine dose across all 80 patients studied, including 59 patients on a high dose, was 0.4 (0.28–0.7) μg/kg/min. Overall, simultaneous measurement of MAP (mmHg) at the femoral and radial arterial sites produced mean (95% CI) MAP values of 81 (79–83) and 78 (76–80), respectively, with a mean difference of 3.3 (2.67–3.93), *p* < 0.001. In Bland–Altman analysis of MAP measurements, the detected effect sizes were 1.14 and 1.04 for the overall and high-dose cohorts, respectively, which indicates a significant difference between the measurements taken at each of the two arterial sites. The Pearson correlation coefficient indicated a weak but statistically significant correlation between MAP gradient and norepinephrine dose among patients receiving a high dose of norepinephrine (*r* = 0.289; *p* = 0.026; 95% CI 0.036–0.508).

**Conclusion:**

In septic shock patients, MAP readings were higher at the femoral site than at the radial site, particularly in those receiving a high dose of norepinephrine.

**Clinical trial registration:**

[ClinicalTrials.gov], identifier [NCT03475667].

## Introduction

Invasive blood pressure (IBP) monitoring is a common procedure carried out in critically ill patients admitted to the intensive care unit (ICU) to optimize their hemodynamics. The international guidelines of the Surviving Sepsis Campaign (SSC) for the management of sepsis also suggest the placement of an arterial catheter as soon as practical in all patients requiring vasopressors (1). The accurate measurement of systolic blood pressure (SBP) and mean arterial pressure (MAP) is essential both for identification of circulatory shock and for maintenance of hemodynamic targets for better clinical outcomes while minimizing the untoward side effects of vasopressor agents (1–4). The artery most commonly used for catheter placement is the radial artery, and this site is considered to be both easily accessible and safe, with fewer complications arising in comparison to other sites, such as the femoral, dorsalis pedis, posterior tibial, ulnar, or brachial artery (5–8).

Generally, in physiological conditions, systolic blood pressure (SBP) is higher and diastolic blood pressure (DBP) is lower, while mean arterial pressure (MAP) is unchanged, when measurements are taken at a peripheral arterial site (radial) in comparison to a central arterial site (femoral) (9). However, in various clinical conditions, it has been reported that simultaneous MAP measurements taken at the most commonly used sites in each case (the radial artery and the femoral artery) differ significantly. During deep hypothermic cardiac arrest and during high-risk surgeries, including cardiopulmonary bypass and liver transplantation, MAP measurements have been found to be significantly different, with femoral site readings being higher than radial site readings (10–16). There is a possibility that this site-specific difference in IBP measurements might be explained by poorly understood pathophysiological changes, such as regional auto-regulation, atherosclerotic conditions, and tunica media area, and their relationships with the effects of vasopressors (norepinephrine alone or with other vasoactive agents) (17, 18). These studies suggest giving preference to central artery site measurement in high-risk peri-operative and medical patients experiencing circulatory shock requiring vasopressor therapy in order to deliver optimal care through accurate hemodynamic monitoring.

Among extant studies in the septic population, many have reported observing higher MAP values at the femoral artery site (19–23), while others have not (24, 25). However, across all the studies available, MAP measurements have also been included either from patients with non-septic conditions or from those not receiving vasopressors, which might have influenced their results. The available findings of these studies suggest the need for further evidence in the septic shock population, including separate subgroup analysis for patients receiving a high dose of norepinephrine, as this treatment is not uncommon (26), and underestimation of MAP measurements at the radial artery site could lead to unnecessarily high-dose vasopressor therapy and its related complications, including arrhythmia and digital or limb necrosis (27–29). This is especially important in septic shock patients, to whom vasopressors are frequently administered for an extended period of time.

Our study aimed to determine the MAP gradient between the femoral and radial artery sites in septic shock patients receiving norepinephrine (≥0.1 μg/kg/min), with pre-planned subgroup analysis for those receiving a high dose of norepinephrine (≥0.3 μg/kg/min).

## Materials and methods

### Study design

This prospective observational study was conducted at a tertiary care center, specifically the 20-bed ICU of a university hospital in India, from Apr 2018–Jan 2020. The primary objective of this study was to compare femoral and radial arterial invasive blood pressure measurements in patients receiving norepinephrine (≥0.1 μg/kg/min) for septic shock and to correlate the pressure gradient with the dose of norepinephrine. The study protocol was approved by the Institutional Ethics Committee (IEC code: 2018-27-DM-EXP). A waiver of consent was granted by the Ethics Committee. The study was recorded on the ClinicalTrials.gov website (ClinicalTrials.gov identifier: NCT03475667).

### Study population

Critically ill adult septic shock patients (as per Sepsis-3 definition) requiring norepinephrine infusion (≥0.1 μg/kg/min) and in whom the site of arterial invasive blood pressure monitoring was changed from radial to femoral, due to the current practice in our ICU as per the existing literature, were considered for inclusion in this study. All consecutively presenting eligible patients were included if they received a static dose of norepinephrine for at least 30 min with a functional radial artery monitoring system in place. Patients below the age of 18 years, pregnant women, patients with abdominal compartment syndrome, patients in whom a supine position was not feasible due to their clinical condition, and patients with a history of peripheral artery disease were all excluded from the study.

### Data collection

All relevant demographic details, clinical characteristics, all vasopressor doses, and each patient’s need for mechanical ventilation were recorded, along with scores on two measures of ICU severity, namely the Sequential Organ Failure Assessment (SOFA) and the Acute Physiology and Chronic Health Evaluation II (APACHE II). In addition to IBP (SBP, DBP, and MAP) readings, we also noted the number of patients in whom an absolute pressure gradient of ≥5 and ≥10 mm Hg was observed.

### Measurements of blood pressure

In each participating patient, a 16-gauge single-lumen arterial catheter (SLR 16 GA 8’ Arrow international, C.R.a.s. Jamska 2359/47) was used for femoral artery cannulation, while a 20-gauge catheter (BD Venflon™ Pro IV Cannula) was used for the radial artery. The arterial lines (pressure monitoring lines) used at both sites were similar. Blood pressure measurements were recorded in a supine position in cases in which both procedures were carried out on the same side (left or right), with the radial arterial line transducer at the level of the 5th rib in the mid-axillary line (phlebostatic axis), and the femoral line transducer placed at the same level as the radial line transducer. The equality of the levels of both the transducers was confirmed *via* the spirit level technique (30). The adequacy of damping was assessed by the fast-flush test.

All pressure values (SBP, DBP, and MAP) were recorded simultaneously three times within a 5-minute period, by freezing or taking a snapshot of the monitor, after placement of the femoral arterial catheter and before removal of the radial arterial line. The three readings for each value were averaged for analysis purposes.

### Sample size

In a previous observational study, the minimum average paired difference in MAP (mean ± SD) between radial (86.7 ± 10.7) and femoral (91.1 ± 11.5) site blood pressure recordings was 4.4 ± 11.1 mmHg (effect size = 0.396) (22). Based on this effect size, with a two-sided 95% confidence interval and 80% power for the study, the estimated sample size required with paired groups was 53. In our study, we also planned to carry out a subgroup analysis in patients receiving a high dose of norepinephrine (≥0.3 μg/kg/min). Therefore, we included 80 patients. Sample size was estimated using the G*Power software package, version 3.1.9.2 (Düsseldorf University, Germany).

### Statistical analysis

The normality of the continuous variables were assessed; variable was considered to be normally distributed when the Z score of the skewness was within a range of ±3.29 (31). Normally distributed data are reported in the form of means (95% confidence interval, CI); other data are presented in the form of medians (interquartile range, IQR). Categorical variables are reported as numbers (percentage). A paired sample *t*-test was used to test the significance level of the mean differences observed. A Bland–Altman analysis was conducted, in which the bias (mean difference between two paired measurements) and corresponding 95% limits of agreement (reported in the form of mean ± 1.96 SD for the paired differences) were calculated for both the overall group and the high-dose group. To evaluate the absolute change in mean values in paired observations with respect to the corresponding pooled standard deviation, the effect size of the mean difference was calculated. The Pearson correlation coefficient (*r*) was used as an index of the relationship between norepinephrine dose and blood pressure gradient. All statistical analyses were performed using SPSS for Windows, version 23.0 (SPSS Inc., Chicago, IL, USA), and a two-tailed *P*-value < 0.05 was considered to represent statistical significance.

## Results

**FIGURE 1:**
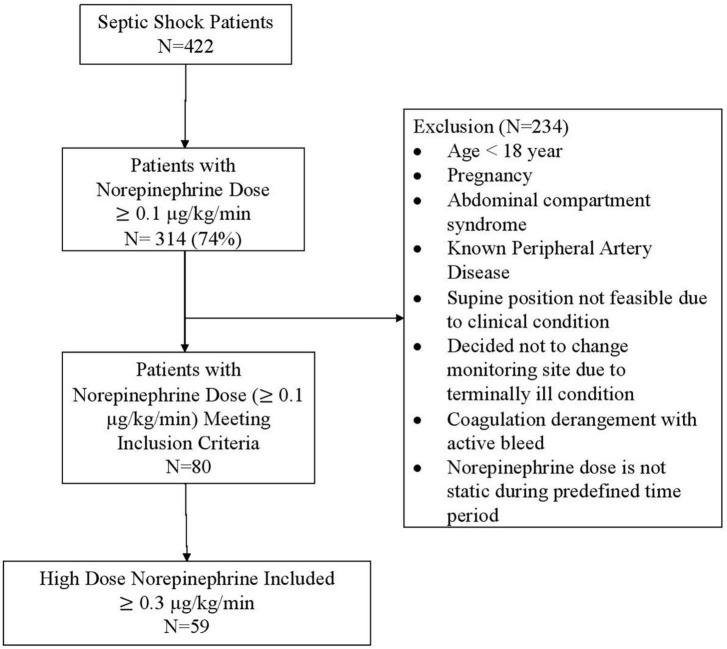
Patients included in the study.

During the study period, a total of 520 patients were admitted to the ICU, and 422 of these received vasopressor therapy for septic shock. Among septic shock patients, 314 (74%) received a norepinephrine dose of at least 0.1 μg/kg/min at any point during their ICU stay (Figure 1). In accordance with the inclusion and exclusion criteria, 80 patients were included in this study, among whom the median norepinephrine dose was 0.4 (0.28–0.7) μg/kg/min. Of these 80 patients, 21 were receiving a norepinephrine dose of 0.1–0.29 μg/kg/min, while 59 were receiving ≥0.3 μg/kg/min. Across all included patients, the median age was 46.5 (31–56) years and the median APACHE II score at ICU admission was 23 (18–29). On the day of study inclusion, the median SOFA score was 15.5 (12–18), 66 patients (82.5%) were on mechanical ventilation, and none of the patients were receiving inotropic medication, including dobutamine (Table 1).

**TABLE 1:** Demographic and clinical characteristics of patients included in the study (*n* = 80).

Demographic and clinical characteristics	Value, number, or median (% or IQR)		
	**All patients (≥0.1 μg/kg/min) (*n* = 80)**	**Patients receiving norepinephrine ≥0.1 and <0.3 μg/kg/min (*n* = 21)**	**Patients receiving norepinephrine≥0.3 μg/kg/min (*n* = 59)**
Age (years)	46.5 (31–56)	46.1 (33–55)	45.5 (33–56)
Male, *n* (%)	47 (58%)	12 (56%)	34 (58%)
BMI (kg/m^2^)	22.7 (22–25)	23 (22–26)	22.9 (21.5–25)
APACHE II score at admission, median (IQR)	23 (18–29)	22 (17–26)	24 (19–29)
SOFA score at admission, median (IQR)	11 (9–13)	10 (8–11)	12 (9–14)
SOFA score on day of study, median (IQR)	15.5 (12–18)	15 (10–16)	16 (12–18)
Mechanical ventilation, *n* (%)	66 (82.5%)	15 (71%)	50 (85%)
Medical, *n* (%)	65 (81%)	16 (76%)	49 (83%)
Norepinephrine dose (μg/kg/min), median (IQR)	0.4 (0.28–0.7)	0.2 (0.19–0.25)	0.5 (0.35–1)
Vasopressin, *n* (%)	63 (78.6%)	4 (19%)	50 (85%)
Hydrocortisone, *n* (%)	57 (71.25%)	4 (19%)	50 (85%)
ICU mortality	18 (17.5%)	3 (14%)	15 (26%)

BMI, body mass index; APACHE II, Acute Physiology and Chronic Health Evaluation II; SOFA, Sequential Organ Failure Assessment; IQR, interquartile range; ICU, intensive care unit.

Across all included patients (*n* = 80), invasive blood pressure readings (mmHg) taken simultaneously at the femoral and radial arterial sites revealed that the mean (95% CI) SBP values were 122 (118–126) mmHg and 119 (115–123) mmHg, respectively, with a statistically significant difference between the means of 2.9 (1.1–4.7), *p* = 0.002; additionally, the mean (95% CI) MAP values were 81 (79–83) mmHg and 78 (76–80) mmHg, respectively, with a statistically significant difference between the means of 3.3 (2.7–3.9), *p* < 0.001. In the subgroup of patients receiving a high dose of norepinephrine (*n* = 59), the mean (95% CI) MAP values (mmHg) at the femoral and radial arterial sites were 80 (78–83) and 78 (75–80), with a statistically significant difference between the means of 2.9 (0.6–5.2), *p* < 0.001 (Table 2).

**TABLE 2:** Systolic blood pressure (SBP), diastolic blood pressure (DBP) and mean arterial pressure (MAP) at femoral and radial arterial sites in study patient cohort.

Measurement		Femoral artery	Radial artery	Mean difference (95% CI)	*P*-value
SBP (mmHg) (95% CI)	All (*n* = 80)	122 (118–126)	119 (115–123)	2.9 (1.12 to 4.68)	0.0023
	High dose (*n* = 59)	121 (116–125)	118 (114–123)	2.3 (0.7 to 3.9)	0.22
DBP (mmHg) (95% CI)	All (*n* = 80)	61 (59–62)	59 (58–61)	1.4 (0.6 to 3.6)	0.001
	High dose (*n* = 59)	60 (58–62)	59 (57–60)	1.2 (0.5 to 5.2)	0.0054
MAP (mmHg) (95% CI)	All (*n* = 80)	81 (79–83)	78 (76–80)	3.3 (2.67 to 3.93)	<0.0001
	High dose (*n* = 59)	80 (77–82)	77 (75–79)	2.9 (0.63 to 5.17)	<0.0001

Overall, an absolute pressure gradient of ≥5 mmHg (femoral > radial) was observed for SBP in 21 patients (26%) and for MAP in 17 patients (21%). Among the subgroup of patients receiving high-dose norepinephrine, an SBP gradient of ≥5 mmHg was observed in 13 patients (22%), and a MAP gradient of ≥5 mmHg was observed in 11 patients (19%), with the radial site frequently underestimating the central blood pressure (Table 3).

**TABLE 3:** Number of patients having systolic blood pressure (SBP) and mean arterial pressure (MAP) differences between femoral (F) and radial (R) sites, with cut-off values 5 and 10 mm of Hg.

Differences	Overall (*n* = 80)				High dose (*n* = 59)			
Δ (F-R)	ΔSBP ≥ 10	ΔSBP ≥ 5	ΔMAP ≥ 10	ΔMAP ≥ 5	ΔSBP ≥ 10	ΔSBP ≥ 5	ΔMAP ≥ 10	ΔMAP ≥ 5
F > R	12	21	3	17	8	13	2	11
F < R	3	5	0	0	2	3	0	0
Norepinephrine dose, M (IQR)	0.4 (0.28–0.7)				0.5 (0.35–1)			
ΔFR, M (IQR)	2 (−1–5)		3 (2–4)		2 (−1–4)		3 (2–4)	

ΔSBP, systolic blood pressure (SBP) gradient between femoral and radial artery sites; ΔMAP, mean arterial pressure (MAP) gradient between femoral and radial artery sites; M (IQR), median (interquartile range).

**FIGURE 2:**
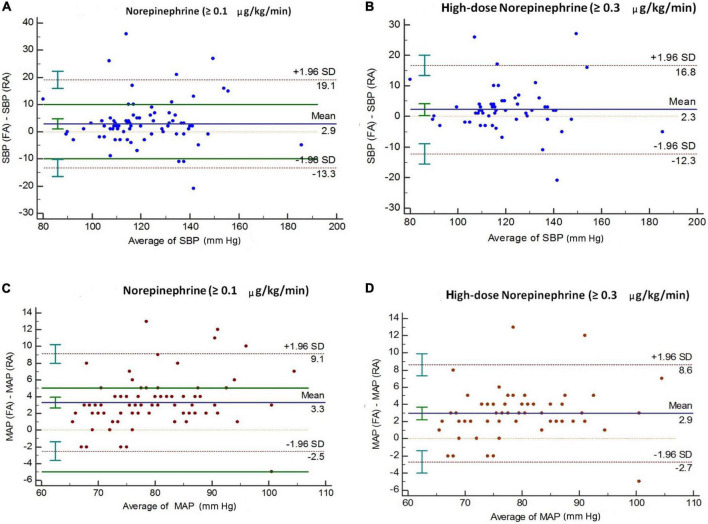
Bland–Altman analysis of differences between femoral and radial arterial pressure among all patients (*n* = 80), and among those on high-dose norepinephrine (*n* = 59). Panels **(A,B)** represent SBP; panels **(C,D)** represent MAP. The solid line represents bias (the mean difference between simultaneous measurements). Dotted lines show 95% limits of agreement (bias ± 1.96 SD). SBP, systolic blood pressure; MAP, mean arterial pressure.

Bland–Altman analysis indicated the presence of marked discrepancies or uncertainty in the measurement between the two methods, as the 95% limits of agreement indicated no specific direction for the change in mean difference (Figure 2 and Table 4). To quantify the absolute change in mean values in paired observations with respect to the corresponding pooled standard deviation, the effect size of the mean difference was also calculated. This showed that the effect sizes for the overall and high-dose cohorts, respectively, were 0.35 and 0.32 for SBP (effect sizes ranging from 0.2 to 0.49 are considered small) and 1.14 and 1.04 for MAP (effect sizes ≥ 0.8 are considered large) (Figure 2 and Table 4).

**TABLE 4:** Bias (mean of the difference) and 95% limits of agreement between arterial pressure measurements at the femoral and radial arterial sites, calculated *via* Bland–Altman analysis accounting for repeated simultaneous measurements.

Norepinephrine group	Arterial pressure (mmHg)	Femoral	Radial	SD	Bias	95% limits of agreement	SE (%)	Effect size
Overall (≥0.1 μg/kg/min) (*n* = 80)	SBP	122 (118–126)	119 (115–123)	8.1	2.9	−13.3 to 19.1	28	0.35
	MAP	81 (79–83)	78 (76–80)	2.9	3.3	−2.5 to 9.1	45	1.14
High dose (≥0.3 μg/kg/min) (*n* = 59)	SBP	121 (116–125)	118 (114–123)	7.3	2.3	−12.3 to 16.8	27	0.32
	MAP	80 (77–82)	77 (75–79)	2.8	2.9	−2.7 to 8.6	48	1.04

Data presented in the form of medians (interquartile range), along with the corresponding bias, SD of the mean difference, 95% limits of agreement, and effect size. SBP, systolic blood pressure; MAP, mean arterial pressure; SD, standard deviation; SE, standard error.

**FIGURE 3:**
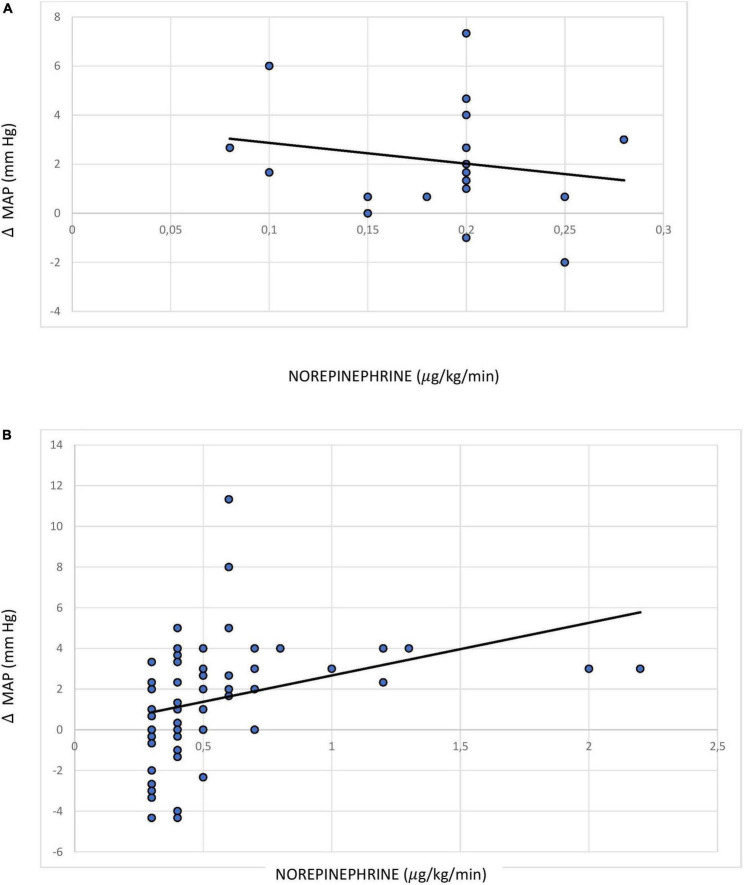
Pearson correlation between norepinephrine dose and mean arterial pressure difference between the femoral and radial sites among patients receiving **(A)** norepinephrine dose <0.3 (0.1–0.29) μg/kg/min; **(B)** norepinephrine dose ≥0.3 μg/kg/min.

The Pearson coefficient representing the correlation between MAP gradient (femoral > radial) and norepinephrine dose was not found to be significant across the entire sample, i.e., among patients receiving a norepinephrine dose ≥0.1 μg/kg/min (*r* = 0.105; *p* = 0.805; 95% CI: −0.118 to 0.317). However, there was a statistically significant weak positive correlation between MAP gradient and norepinephrine dose among patients receiving a high dose of norepinephrine (*r* = 0.289; *p* = 0.026; 95% CI: 0.036 to 0.508) (Figures 3A, B).

## Discussion

Our study found that MAP measurements taken at the radial artery site are frequently underestimates in comparison to those taken at the femoral artery site in septic shock patients requiring norepinephrine (≥0.1 μg/kg/min). In previous studies comparing MAP measurements between the femoral and radial arterial sites in septic shock patients, findings have been inconsistent (Table 5). Studies by Dorman, Compton, Galluccio, Kim, and Wisanusattra have reported significantly higher MAP at the femoral arterial site when measured simultaneously with the radial site (19–23), while those by Mignini and Antal have not observed this (24, 25). Among these, only the studies conducted by Dorman and Kim recruited a cohort of only septic shock patients.

**TABLE 5:** Summary of studies examining differences in mean arterial pressure (MAP) between the femoral and radial sites in critically ill septic shock patients.

References	Number of patients	Study population	Average norepinephrine dose	Bias for femoral–radial ΔMAP (mmHg) [95% CI of agreement]	Statistically significant difference
Dorman et al. (19)	14	Septic shock	86 μg/min	15	Yes
Compton et al. (20)	25	Septic and non-septic shock	0.3 μg/kg/min	3 [−17 to +11]	Yes
Gallucio et al. (21)	24	Septic and non-septic shock	0.26 μg/kg/min	4 [−12 to +4]	Yes
Kim et al. (22)	37	Septic shock	0.1 μg/kg/min	5 [−17 to +7]	Yes
Wisanusattra et al. (23)	32	Majority septic shock (87.5%)	0.85 μg/kg/min	7.5 [−23.7 to 8.6]	Yes
Mignini et al. (24)	55	Septic and non-septic shock	0.1 μg/kg/min	3 [−11 to +5]	No
Antal et al. (25)	71	Sepsis	0.14 μg/kg/min	1.1 [−8.5 to 10.7]	No
Our study	80	Septic shock	0.4 μg/kg/min	3.3 [−2.5 to 9.1]	Yes

The population of the study by Dorman (19) included a small number of septic shock patients (*n* = 14) receiving a mean norepinephrine dose of 86 mcg/min, and the mean femoral site MAP observed was 15 mmHg higher than that observed at the radial artery site. Kim (22) included 37 septic shock patients who were receiving a mean norepinephrine dose of 0.1 μg/kg/min, with multiple measurements taken after inclusion, and found that radial MAP was on average 5 mmHg lower (95% CI: −17 to +7). In a recently published study by Wisanusattra (23), 32 patients (28 with septic shock) who were receiving a mean norepinephrine dose of 0.85 mcg/kg/min showed a MAP gradient between the femoral and radial sites of 7.6 mmHg over multiple hourly serial readings over the course of 24 h. In contrast, the results of the studies conducted by Compton, Galluccio, Mignini, and Antal did not exclusively represent a septic shock cohort, and their findings might have been influenced by the inclusion of non-septic shock patients (Table 5).

In our study, across all septic shock patients included, the median norepinephrine dose was 0.4 μg/kg/min and the mean radial site MAP was 3.3 mmHg lower than the femoral arterial site MAP. More than 20% of patients had a higher MAP (gradient ≥ 5 mmHg) at the femoral site, while no patients had a higher MAP (gradient ≥ 5 mmHg) at the radial artery site. This higher MAP (gradient ≥ 5 mmHg) at the femoral site was also observed in 58% of patients in the Kim study (22) and in 59% of patients receiving a high dose of norepinephrine in the study by Wisanusattra (23). Across all studies, there were either fewer (2–4%) or no patients with MAP readings ≥5 mmHg higher at the radial artery site compared to the femoral site (19–25).

According to the pre-planned analysis conducted in our study in the subgroup of patients receiving high-dose norepinephrine [median (IQR) dose: 0.5 (0.35–1) μg/kg/min], the Pearson correlation coefficient indicated a weak correlation (*r* = 0.289; *p* = 0.026) between MAP gradient and vasopressor dose, which is a similar finding to that of the study by Kim (*r* = 0.33, *p* < 0.001) (22). In another recent study conducted by Wisanusattra, a strong correlation was observed between MAP gradient and vasopressor dose (*r* = 0.89; *p* < 0.0001) (23). This correlation might be relevant for clinical outcomes, as prolonged unnecessary exposure to a higher dose of norepinephrine during septic shock leads to its own complications, such as arrhythmia and digital or limb necrosis. However, we acknowledge that the risk–benefit ratio of giving preference to the femoral arterial site for MAP measurement in patients with septic shock is still not clear and needs to be investigated further. Indeed, a recent study has found that the difference in non-invasive blood pressure readings between the brachial and radial artery correlates with the MAP difference between the femoral and radial artery (32); this could be considered as a means of identifying patients who are candidates for preferential use of the femoral artery site for invasive pressure measurement.

### The limitations and strength of the study

Our study has certain limitations. First, we did not include patients who were receiving a norepinephrine dose <0.1 μg/kg/min. Second, we did not investigate the effects of age, radial artery diameter, intra-thoracic pressure, or intra-abdominal pressure on the MAP gradient between the femoral and radial arterial sites. Additionally, we did not analyze the effect of concurrent use of vasopressin on the MAP gradient observed in patients who were receiving this. The long-term adverse effects of femoral artery site cannulation, such as digital ischemia, thrombosis, or other complications, were not followed up in our study. On the other hand, the primary strength of our study is that we included only septic shock patients receiving a norepinephrine dose of at least 0.1 μg/kg/min, including a pre-planned analysis with an adequate sample size of patients receiving a norepinephrine dose of at least 0.3 μg/kg/min.

## Conclusion

In comparison to the femoral artery site, invasive pressure monitoring at the radial artery site frequently underestimates SBP and MAP in septic shock patients receiving norepinephrine therapy. Hence, the findings of our study suggest that the femoral artery could be considered over the radial artery for accurate MAP measurement in septic shock patients, particularly in those who are receiving a high dose of norepinephrine, in order to minimize possible side effects arising from unnecessary exposure to higher doses of vasopressor agents for a prolonged period of time. However, the risk–benefit ratio of giving preference to the femoral arterial site in these patients is still not clear and needs to be investigated further.

## Data availability statement

The raw data supporting the conclusions of this article will be made available by the authors, without undue reservation.

## Ethics statement

The studies involving human participants were reviewed and approved by the Institutional Ethics Committee, SGPGIMS, Lucknow, India. Written informed consent for participation was not required for this study in accordance with the national legislation and the institutional requirements.

## Author contributions

BB: data curation, investigation, and original draft preparation. MG: conceptualization, methodology, project administration, supervision, validation, and writing – review and editing. PM: methodology, formal analysis, and software. AA: supervision and writing – review and editing. BP: methodology, and writing – review and editing. AB: project administration and writing – review and editing. All authors contributed to the article and approved the submitted version.
